# Supplementary material for: The molecular basis of Human FN3K mediated phosphorylation of glycated substrate
Source: bioRxiv. 2024 Aug 5:2024.08.05.606604. Preprint. [Version 1] doi: 10.1101/2024.08.05.606604 (PMC11326186; doi:10.1101/2024.08.05.606604)
Supplement: Supplement 1 [file media-1.pdf]

Supplemental figures and tables

The molecular basis of Human FN3K mediated phosphorylation of glycated substrate

Ankur Garg[1,2,3 #], Kin Fan On[1,2,3 #], Yang Xiao[4,5], Elad Elkayam[1,2,*], Paolo Cifani[2], Yael David[4,5] and Leemor Joshua-Tor[1,2,3,6].

[1] W. M. Keck Structural Biology Laboratory, [2]Cold Spring Harbor Laboratory, One Bungtown Road, Cold Spring Harbor, New York, 11724 USA

[3] Howard Hughes Medical Institute, Cold Spring Harbor Laboratory, One Bungtown Road, Cold Spring Harbor, New York, 11724 USA

[4] Chemical Biology Program, Memorial Sloan Kettering Cancer Center, New York, NY, USA

[5] Tri-Institutional PhD Program in Chemical Biology, New York, NY, USA

# Equal contribution

[*] Current Address- Ventus Therapeutics, Waltham, Massachusetts, 02453, USA

[6] Corresponding author leemor@cshl.edu

Figure S1-

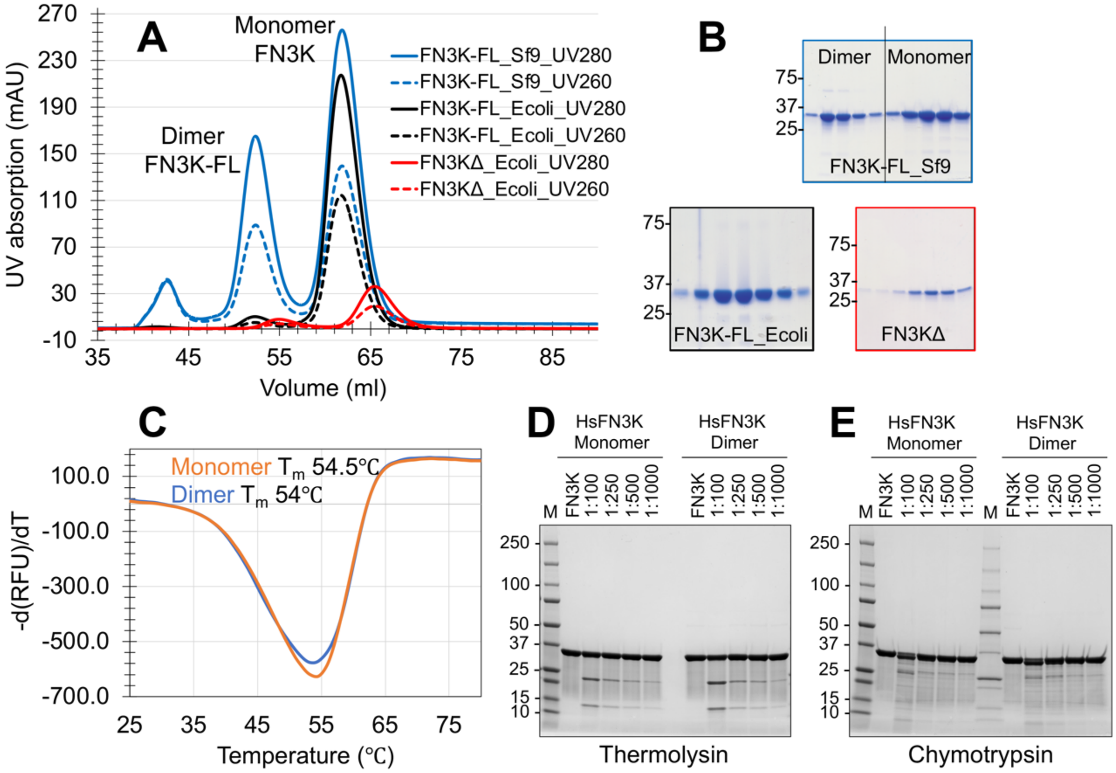

Figure S1- Purification and biophysical characterization of HsFN3K. (A) SEC chromatogram of HsFN3K purified from different expression sources. FN3K expressed in Sf9 cells showed a significant dimeric peak in SEC. (B) SDS-PAGE showing different purified HsFN3K proteins. (C) Thermal melting spectra for the dimeric and monomeric HsFN3K (expressed in Sf9) species. The melting temperature (Tm) for both species are very similar, suggesting a similar structural fold for both. Limited proteolysis of HsFN3K (Sf9) monomer and dimer using (D) Thermolysin and (E) Chymotrypsin proteases. The w/w ratio of protein to protease is noted at the top of each lane. “M” is a protein ladder.

Figure S2-

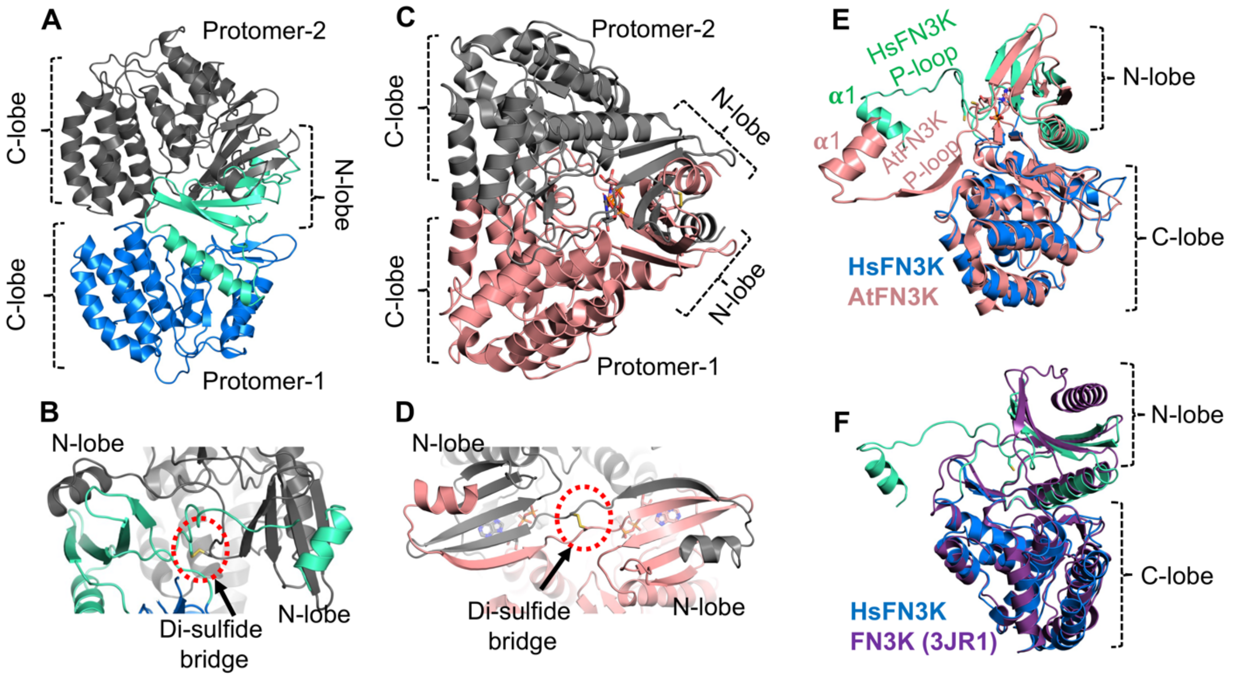

Figure S2- A comparison of HsFN3K and AtFN3K crystal structures. (A) Cartoon representation of the HsFN3K dimer in the asymmetric unit in the apo-HsFN3KΔ crystal structure. (B) The P-loop Cys24 (shown in sticks) disulfide bridge in the HsFN3KΔ dimer. (C) Cartoon representation of AtFN3K dimer as observed in the asymmetric unit. (D) The P-loop Cys (shown in sticks) disulfide bridge in the AtFN3K structure. (E) Superposition of the HsFN3K and AtFN3K (PDBid 6OID) structures showing the difference in P-loop orientations. (F) Structure superposition of HsFN3K and *E. coli* FN3K (PDB 3JR1) illustrating the difference in P-loop orientation in the two structures.

Figure S3-

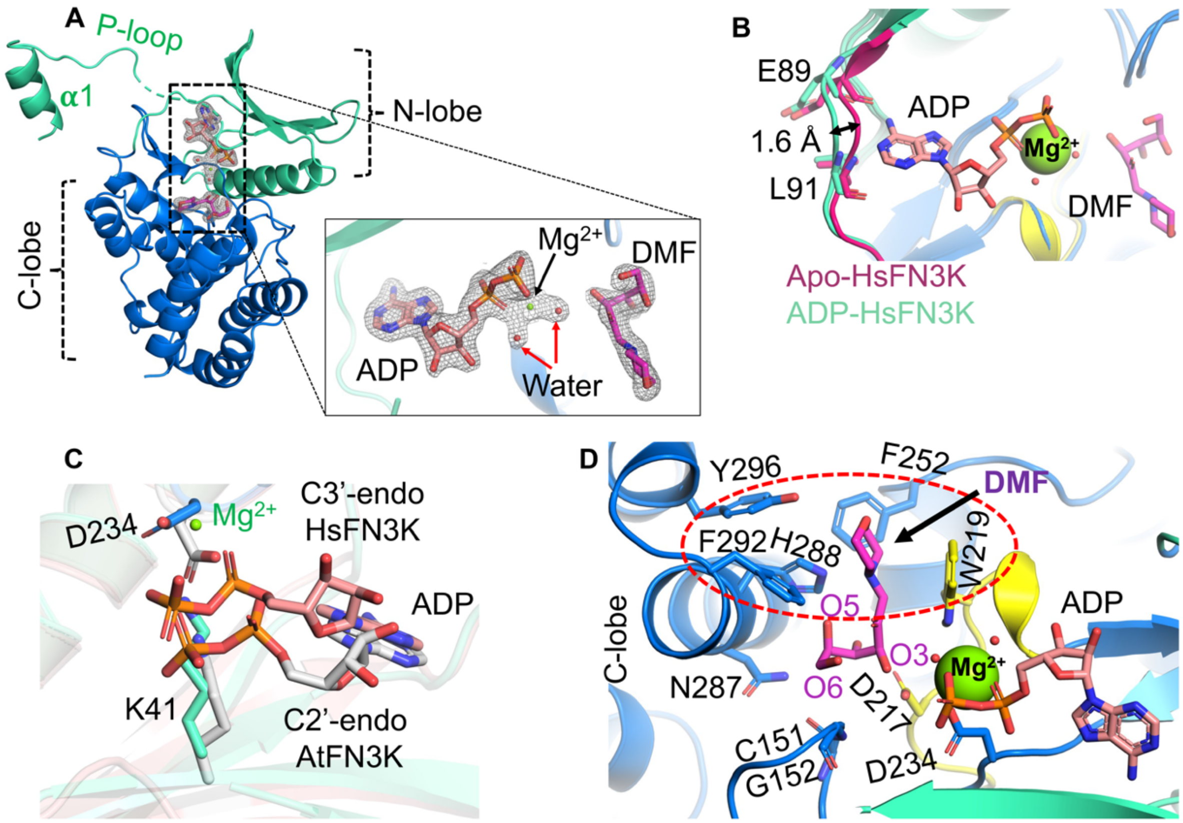

Figure S3- (A) Cartoon representation of the crystal structure of HsFN3K bound to ADP-DMF-Mg^{2+} (protomer-1). The 2Fo-Fc electron density observed for ADP, DMF, and Mg^{2+} is shown. Two water molecules coordinating the Mg^{2+} ion is also visible in the electron density map. The dotted section in the P-loop represents the unmodelled region due to missing electron density (B) A close-up view of the superposed nucleotide-sensing loop in apo-HsFN3K (red) and ADP-HsFN3K (light teal) showing the subtle adjustment upon nucleotide binding. The residues involved in direct interactions with the adenine base (E89 and L91) are shown as sticks, and the loop movement is also marked. (C) ADP nucleotide binding in the HsFN3K and AtFN3K structures. The sugar geometry in HsFN3K is canonical C3'-endo, while in AtFN3K is likely in an C2'-endo conformation, resulting in a clear difference in ADP placement in the catalytic site. The C3'-endo geometry in HsFN3K favors the interaction of the nucleotide phosphates with Lys41. (D) The DMF substrate binding site in HsFN3K (shown with red dotted circle) is lined with several aromatic residues (shown as sticks). The sugar interacts directly with D217 via its phosphate-receiving O3' atom.

Figure S4-

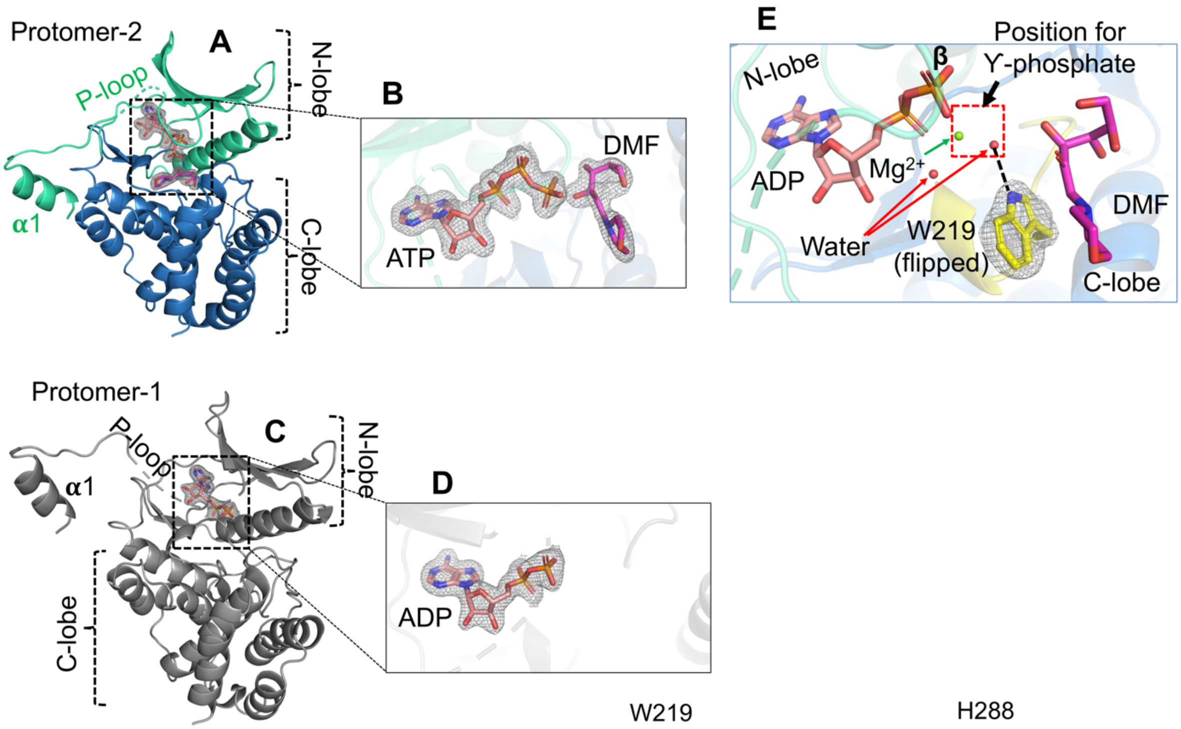

F

			W219 ↓		H288 ↓	
Homo sapiens (FN3K)	211	PALLHGDLWSGNVAE	.....	YQLFNYLNHWNHFGRE	295
Homo sapiens (FN3KRP)	211	PALLHGDLWGGNVAE	.....	YQLFHYLNHWNHFGSG	295
Mus musculus	211	PALLHGDLWSGNVAE	.....	YQLFNYLNHWNHFGRE	295
Bos taurus	211	PALLHGDLWSGNVAE	.....	YQLFNYLNHWNHFGRQ	295
Canis familiaris	293	PALLHGDLWSGNVAE	.....	YQLFNYLNHWNHFGQG	377
Gallus gallus	225	PALLHGDLWAGNVAE	.....	YQLFNYINHWNHFGTG	309
Arabidopsis thaliana	224	PCLLHGDLWSGNIAY	.....	YLLYHYLNHYNLFGSG	308
Lactiplantibacillus plantarum	183	PSLLHGDLWSGNYLF	.....	YQLYYLLAHLNLFGET	268
Staphylococcus aureus	185	PSLLHGDLWGGNYMF	.....	YRLYLLMVHLLKFGEM	270

Figure S4- Structural features of HsFN3K bound with ATP and DMF (A) Cartoon representation of protomer-2 in the HsFN3K-ATP-DMF structure, with ATP and DMF shown as sticks. (B) 2Fo-Fc electron density for the ATP and the DMF shown at a 1σ level with a carved radius of 1.6 Å. (C) Cartoon representation of protomer-1 in HsFN3K-ATP-DMF structure, showing only ADP (in sticks) bound in the catalytic site and (D) the observed 2Fo-Fc electron density for ADP shown at a 1σ level with a carved radius of 1.6 Å. No density for DMF was observed in the substrate binding site. (E) Close-up view of the catalytic site in the HsFN3K-ADP structure showing ADP and DMF in sticks. The Mg^{2+} and water molecule occupies the position of the γ-phosphate (shown as a red dotted square) which allows W219 (yellow sticks with 2Fo-Fc density shown at a 1σ level) to flip and interact with the water molecule (shown as a black dotted line). (F) The sequence alignment for FN3K shows the conservation of W219 and H288 across different species.

Figure S5-

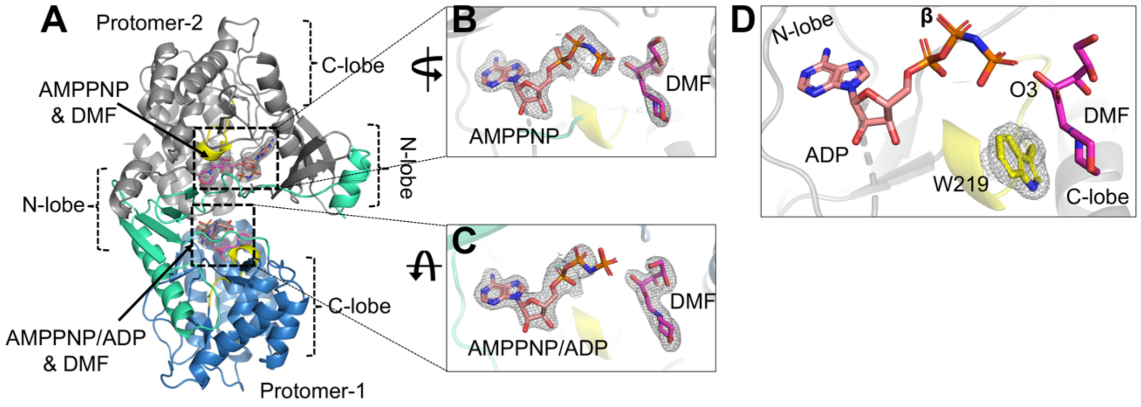

Figure S5- The crystal structure of HsFN3KΔ-AMPPNP-DMF shown in (A) cartoon representation. The AMPPNP and DMF observed in each protomer are shown in sticks with the superimposed 2Fo-Fc electron density shown at a 1σ level. The close-up view of the catalytic site in (B) protomer-2 and (C) protomer-1 are shown. The density for the γ-phosphate in protomer-1 is not very clear, but could still be built as AMPPNP. (D) AMPPNP binding in the protomer-2 catalytic site is unable to induce W219 flipping. The W219 is shown in predominantly the alternative conformation (as yellow sticks) with 2Fo-Fc electron density shown at 1σ level with a carved radius of 1.6 Å.

Figure S6-

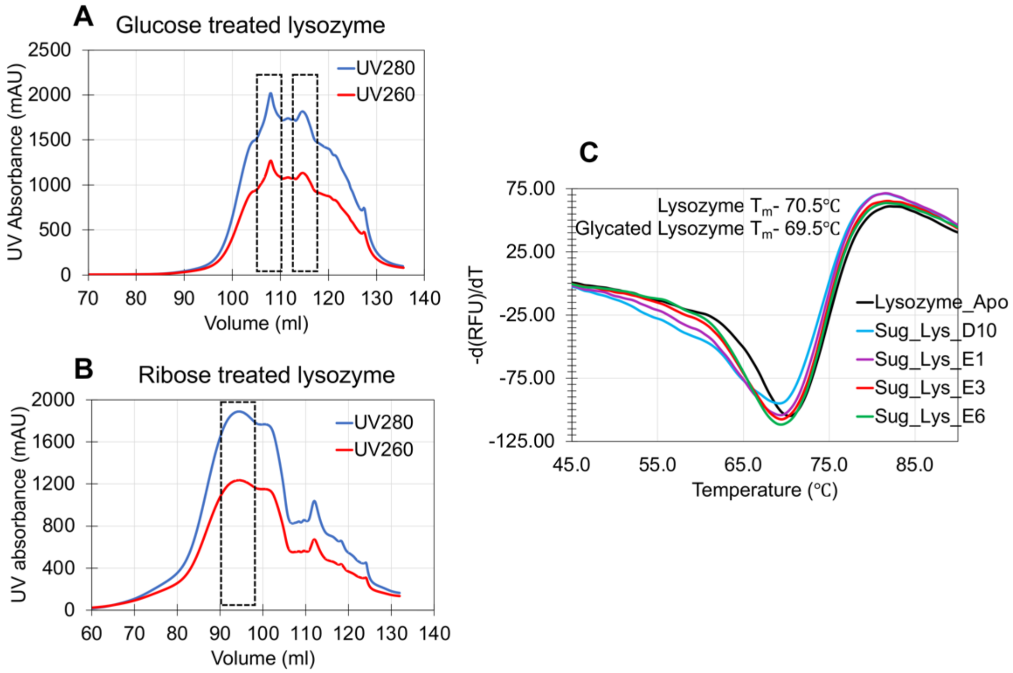

Figure S6- SEC chromatograms for the glycated lysozymes incubated with (A) glucose and (B) ribose sugars for 21 and 7 days, respectively. Glycated lysozyme was distributed in different peaks in both treatments and was verified by MS analysis. The dotted black box around the peaks shows the used glycated lysozyme fractions in the assays (C) The thermal melting spectra for the glycated lysozyme (with either glucose or ribose) demonstrate that the Tm following the sugar treatment did not change.

Figure S7-

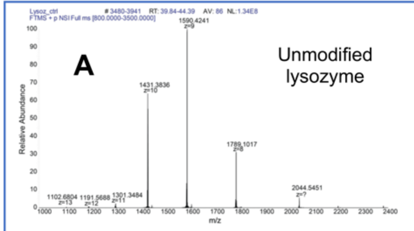

z	Peak 1	Glucose added	0
7	2044.545		14304.765
8	1789.102		14304.755
9	1590.424		14304.751
10	1431.384		14303.763
11	1301.348		14303.752
12	1191.569		14286.738
13	1102.68		14321.75
		Average (Da)	14295.77

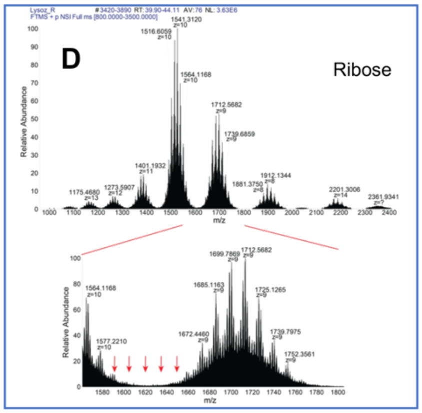

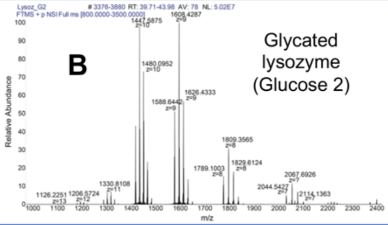

z	Peak 1	Peak 2	Peak 3	Peak 4	Peak 5	Glucose added	0	1	2	3	4
7	2044.543	2067.693	2090.7	2114.136	2137.287		14304.75	14466.8	14627.85	14791.9	14953.96
8	1789.1	1809.357	1829.612	1849.744	1870.001		14304.74	14466.79	14628.84	14789.89	14951.95
9	1590.424	1608.429	1626.433	1644.439	1662.334		14304.75	14466.79	14628.83	14790.88	14951.94
10	1431.483	1447.687	1463.891	1480.095	1496.301		14304.76	14466.8	14628.83	14790.88	14952.94
11	1301.438	1316.17	1330.811	1345.543	1360.275		14304.74	14466.79	14627.84	14789.89	14951.94
12	1192.985	1206.572	1220.077	1233.665	1247.086		14303.73	14466.78	14628.84	14791.89	14952.94
13	1101.371	1113.836	1126.225	1138.691			14304.73	14466.78	14627.83	14789.88	
						Average (Da)	14295.73	14457.79	14619.83	14781.87	14943.93
						**Δ-mass (Da)**	**0**	**162.06**	**162.04**	**162.04**	**162.06**

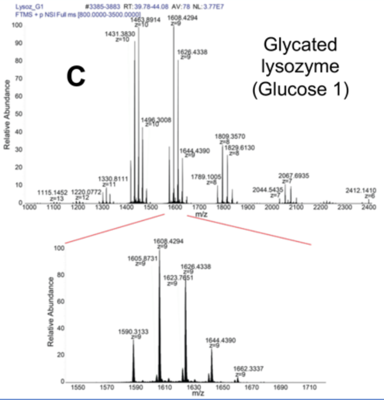

z	Peak 1	Peak 2	Peak 3	Peak 4	Peak 5	Glucose added	0	1	2	3	4
7	2044.545	2067.694	2090.7	2113.993	2137.287		14304.77	14466.8	14627.85	14790.9	14953.96
8	1789.101	1809.357	1829.613	1849.744	1870.251		14304.75	14466.8	14628.85	14789.9	14953.95
9	1590.313	1608.429	1626.434	1644.439	1662.334		14303.75	14466.8	14628.84	14790.89	14951.94
10	1431.383	1447.688	1463.892	1480.096	1496.301		14303.76	14466.81	14628.84	14790.88	14952.94
11	1301.347	1316.079	1330.811	1345.543	1360.548		14303.74	14465.79	14627.84	14789.89	14954.95
12	1192.984	1206.656	1220.077	1233.665	1246.919		14303.73	14467.79	14628.84	14791.89	14950.94
13	1101.371	1113.837	1126.302	1138.768	1151.234		14304.73	14466.78	14628.84	14790.88	14952.94
						Average (Da)	14295.73	14457.8	14619.84	14781.88	14943.93
						**Δ-mass (Da)**	**0**	**162.07**	**162.04**	**162.04**	**162.05**

Figure S7- The intact mass Mass-Spectrophotometry (MS) analysis for (A) unmodified lysozyme and (B-C) glucose glycated lysozymes. Thermo QualBrowser calculated mass difference (Δmass) and added glucose molecules are listed in respective tables next to the spectra (D) Intact MS analysis for ribose-modified lysozyme.
