# Supplementary material for: The molecular basis of Human FN3K mediated phosphorylation of glycated substrate
Source: bioRxiv. 2024 Aug 5:2024.08.05.606604. Preprint. [Version 1] doi: 10.1101/2024.08.05.606604 (PMC11326186; doi:10.1101/2024.08.05.606604)
Supplement: Supplement 2 [file NIHPP2024.08.05.606604v1-supplement-2.pdf]

bioRxiv preprint doi: https://doi.org/10.1101/2024.08.05.606604; this version posted August 5, 2024. The copyright holder for this preprint (which was not certified by peer review) is the author/funder, who has granted bioRxiv a license to display the preprint in perpetuity. It is made available under aCC-BY 4.0 International license.

Table-S1- Summary of X-ray data collection and structure refinement statistics for different crystal structures of *Hs*Fn3K.

	**Apo**	**AMPPNP-DMF**	**ADP-DMF (I)**	**ATP-DMF**	**ADP-DMF (II)**	**FN3K (D217S) ATP**
PDBid	9CX8	9CXN	9CXV	9CXM	9CXW	9CXO
Beamline	ID-24E	ID-24E	AMX-17ID-1	ID24-E	ID24-E	ID24-E
Wavelength (Å)	0.979180	0.979180	0.92011	0.979180	0.979180	0.979180
Data collection on	111920	111920	030421	111021	111021	030422
Space group	$P2_12_12_1$	$P2_12_12_1$	$P2_12_12_1$	$P2_12_12_1$	$P2_12_12_1$	$P2_12_12_1$
Cell dimensions						
a, b, c (Å)	52.35, 111.53, 132.60	52.60, 111.52, 132.91	52.80, 112.00, 132.90	52.93, 112.28, 132.80	52.85, 111.76, 132.66	52.31, 112.45, 132.93
α, β, γ (°)	90, 90, 90	90, 90, 90	90, 90, 90	90, 90, 90	90, 90, 90	90, 90, 90
Resolution (Å)	132.60-1.67 (1.70-1.67)	132.9-1.90 (1.94-1.90)	29.72-1.80 (1.85-1.80)	132.80-1.76 (1.87-1.76)	132.66-1.80 (1.91-180)	132.9-2.32 (2.44-2.32)
No of reflections	295,124	231,201	503,659	441,699	553,663	156,265
Unique reflections	90,249	62,294	73,993	79,306	73,858	34,801
R_{merge} (%)	4.7 (73.9)	4.3 (29.9)	5.6 (63.7)	4.3 (47.9)	5.8 (110)	9.7 (67.7)
<I/σ(I)>	15.8 (1.6)	18.5 (3.6)	18.00 (2.40)	23.2 (3.1)	19.2 (1.6)	12.0 (2.2)
$CC_{1/2}$	0.99 (0.57)	0.99 (0.93)	0.99 (0.85)	0.99 (0.89)	99.9 (75.7)	0.99 (0.73)
Completeness (%)	99.1 (96.0)	99.6 (98.8)	99.9 (98.3)	99.8 (98.2)	99.8 (96.4)	99.3 (96.3)
Multiplicity	3.3	3.8	6.8	5.6	7.5	4.5
**Refinement**						
R_{work}/R_{free}	0.174/0.205	0.176/0.221	0.177/0.205	0.177/0.208	0.187/0.220	0.200/0.237
**No of atoms**						
Protein	4616	4551	4568	4579	4589	4597
Hetero atoms	119	151	152	150	116	129
Solvent atoms	593	537	527	534	388	173
**RMSD**						
Bond lengths (Å)	0.017	0.020	0.018	0.017	0.014	0.002
Bond angles (°)	1.455	1.577	1.559	1.35	1.30	0.565
**Ramachandran statistics**						
Most favored (%)	97.6	97.7	96.8	96.8	97.0	97.4
Allowed (%)	2.4	2.3	3.2	3.2	2.9	2.4
Outliers (%)	0.1	0.0	0.3	0.0	0.0	0.2
Rotamer outlier (%)	0.5	0.0	0.22	0.2	0.2	0.6

bioRxiv preprint doi: https://doi.org/10.1101/2024.08.05.606604; this version posted August 5, 2024. The copyright holder for this preprint (which was not certified by peer review) is the author/funder, who has granted bioRxiv a license to display the preprint in perpetuity. It is made available under aCC-BY 4.0 International license.

[a]Values in parentheses represent the highest resolution shell.
